# Living on the edge: a review on potential risk factors for suicide in adult attention-deficit/hyperactivity disorder

**DOI:** 10.1192/j.eurpsy.2022.485

**Published:** 2022-09-01

**Authors:** J. Miranda, M. Barbosa, A. Tarelho, R. Guedes

**Affiliations:** Centro Hospitalar de Leiria, Department Of Mental Health And Psychiatry, Leiria, Portugal

**Keywords:** Suicide, adhd, risk factors

## Abstract

**Introduction:**

Attention-deficit/hyperactivity disorder (ADHD) is a neurodevelopmental disorder characterized by symptoms including inattention, hyperactivity and/or impulsivity that commonly persists into adulthood. Suicide is a major cause of death in adult ADHD (aADHD) patients. Suicidality is higher in these patients, in possible relationship to various clinical and socio-demographic factors.

**Objectives:**

To review the current literature concerning potential risk factors for suicide in aADHD patients.

**Methods:**

A research was made using the Medline database through the Pubmed search engine, with the following keywords: “adhd”, “suicide”, “risk factors”.

**Results:**

Comorbid psychiatric disorders (major depressive disorder, sleep disturbances, behavior disorders and addictive disorders) are powerful predictors of suicidal behavior in aADHD. Depression is the most frequent diagnosis among aADHD patients with previous suicide attempts. Subtype (mostly the combined type) and severity of ADHD were also associated with a higher number of prior suicide attempts. Impulsiveness, poor emotional self-regulation, recklessness, persistent hyperactivity, inability to relax, engagement in risk behavior (often found in ADHD), common personality traits in aADHD like low frustration tolerance, maladaptive coping and poor problem-solving, as well as interpersonal relationship problems, were associated with higher suicidality. Financial distress caused by unemployment is associated with higher suicidal behaviors. The suicidality is higher in females, mostly associated to self-concept, whereas in males is typically related to impulsivity.

**Conclusions:**

Clinicians should be aware of the potential risk factors for suicide in aADHD patients because the early detection of these factors is fundamental to improve the patients’ quality of life and could contribute to the design of more effective treatments.

**Disclosure:**

No significant relationships.

